# Understanding the social determinants of child mortality in Latin America over the last two decades: a machine learning approach

**DOI:** 10.1038/s41598-023-47994-w

**Published:** 2023-11-27

**Authors:** Carlos Chivardi, Alejandro Zamudio Sosa, Daniella Medeiros Cavalcanti, José Alejandro Ordoñez, Juan Felipe Diaz, Daniela Zuluaga, Cristina Almeida, Edson Serván-Mori, Philipp Hessel, Ana L. Moncayo, Davide Rasella

**Affiliations:** 1 Centre for Health Economics (CHE), University of York, York, UK; 2 School of Psychology, National Autonomous University of Mexico (UNAM), Mexico City, Mexico; 3 Institute of Collective Health (ISC), Federal University of Bahia (UFBA), Salvador, Bahia Brazil; 4 Institute of Global Health (ISGlobal), Barcelona, Spain; 5 Alberto Lleras Camargo School of Government, Universidad de los Andes, Bogota, Colombia; 6 Department of Public Health and Epidemiology, Swiss Tropical and Public Health Institute, Basel, Switzerland; 7 Centro de Investigación para la Salud en América Latina (CISeAL), Pontificia Universidad Católica del Ecuador, Quito, Ecuador; 8 National Institute of Public Health (INSP), Cuernavaca, Mexico

**Keywords:** Risk factors, Public health

## Abstract

The reduction of child mortality rates remains a significant global public health challenge, particularly in regions with high levels of inequality such as Latin America. We used machine learning (ML) algorithms to explore the relationship between social determinants and child under-5 mortality rates (U5MR) in Brazil, Ecuador, and Mexico over two decades. We created a municipal-level cohort from 2000 to 2019 and trained a random forest model (RF) to estimate the relative importance of social determinants in predicting U5MR. We conducted a sensitivity analysis training two more ML models and presenting the mean square error, root mean square error, and median absolute deviation. Our findings indicate that poverty, illiteracy, and the Gini index were the most important variables for predicting U5MR according to the RF. Furthermore, non-linear relationships were found mainly for Gini index and U5MR. Our study suggests that long-term public policies to reduce U5MR in Latin America should focus on reducing poverty, illiteracy, and socioeconomic inequalities. This research provides important insights into the relationships between social determinants and child mortality rates in Latin America. The use of ML algorithms, combined with large longitudinal data, allowed us to evaluate the effects of social determinants on health more carefully than traditional models.

## Introduction

Infant and child mortality are defined as death in the first years of life, they are one of the leading indicators of population health and is distributed unequally worldwide, at the same time, are considered one of the most sensitive health indicators of social and material conditions^1^. Sustainable Development Goal 3.2 calls on all countries to aim to reduce neonatal mortality to at least 12 per 1000 live births and under-five mortality to at least 25 per 1000 live births^2^, by redoubling their efforts to reduce health equity gaps. According to the World Bank, Latin America and the Caribbean has reduced under-five mortality from 33 per 1000 children in 2000 to 16 in 2020. However, there is still great disparity among these countries, as some countries such as Chile or Argentina have achieved a reduction of less than 25 deaths per 1000 children and other countries such as Haiti or Dominica exceed 35 deaths per 1000 children.

While child mortality rates have declined in most countries in recent decades, this decrease has not been equal in terms of global comparisons and racial/ethnic, rural–urban, and socioeconomic disparities. Thus, social determinants of health may be extremely important in understanding such disparity. Social determinants of health can be defined as the conditions in the place where people live, work, and play that can affect health outcomes^3^. Risk and protective social determinants of health that occur during critical and sensitive periods of life can have a greater impact on individuals and lead to multiple diseases and health outcomes. The social determinants of health in under-five child mortality can be evaluated at the individual, interpersonal, organizational, and community levels. Within the individual determinants, variables such as drug use, employment, education, maternal conditions, health behaviors, etc., have been considered. At the interpersonal level, the parental environment, and variables such as social capital have been studied. At the organizational level, the most used variables are those related to access to medical services and at the community level the main variables studied have been the concentration of poverty, pollution, inequality, and public health spending^3^.

Some studies have documented that the under-five child mortality is negatively related to literacy rate and factors associated with healthcare access^4^. Other studies have showed that infant mortality is positively related to living in rural areas due to socioeconomic disadvantage and limited access to medical care^5^. Although multiple social determinants of health at various levels have been systematically associated with infant mortality, most studies have focused mainly on developed countries such as the United States and Europe^3,6^. However, in Latin America and the Caribbean, it has been found that infant mortality may be influenced by favorable socioeconomic conditions in women^7^ diverse living conditions such as sanitation and access to water^8^ poverty^9^ or income level and inequality^10^. To understand how different social determinants impacted child health, Reno and Hyder^3^ proposed a social ecological model by level of organization, we used this theoretical model to understand how different social determinants can affect child mortality. In this way, there is sufficient evidence to think that poverty, inequality, education, access to health resources, and home conditions can be fundamental social determinants to predict infant child mortality in Latin America.

In recent decades, Latin America has shown a significant decrease in infantile mortality mainly due to the progress in the implementation of cost-effective strategies to reduce preventable deaths, such as the increase in primary health care and the improvement of structural and sanitary conditions in homes^11^. Despite this, there are few studies that evaluate the impact of social determinants of health such as poverty, inequality, literacy, access to services such as water and drainage, and indicators of access to medical care on under-five child mortality. In addition, there are no studies that take these variables into account over long periods.

In recent years there has been a growing interest in using machine learning algorithms to try to understand the social determinants of health. Compared to traditional linear models, machine learning models can automatically identify interactions and find the linear and nonlinear relationship between the target variable and the independent variables^12^. The use of machine learning algorithms to extract knowledge from large amounts of data can open new avenues of understanding the complex relationships between social determinants and health outcomes. A few algorithms have been used to understand the relationship between social determinants and infant mortality or under-five child mortality^12–15^. There is a few evidence in Latin America, to our knowledge, extensively examined the impact of social determinants of health on child well-being using longitudinal data spanning the last two decades. In addition, all studies that used machine learning to explore the relationship between under-five child mortality or infant mortality and social determinants used survey data, and none have ever used information from multiple countries during a period of overall socioeconomic improvement of their societies. Assessing social determinants in multiple countries will allow finding the underlying associations between social determinants and child mortality independently of social and political differences.

## Methodology

Our objective was to explore the relationships of social determinants of health in the under-five child mortality in municipalities of Ecuador, Brazil and Mexico from 2000 to 2019.

### Study design

We used a multinational mixed ecological design using longitudinal data with study units at the municipal level in Brazil and Mexico and provinces for Ecuador, which are political-administrative regions that can contain one or several localities but are governed by a local government. Specifically, Mexico and Brazil follow a federal structure, where the country is divided into states. At the second level of observation, we focused on the administrative divisions within each state. Ecuador, however, is not a federal state. It is organized into provinces. Our study focused on the administrative divisions at the second level within each province for Ecuador (called “cantón”).

We used multiple aggregate data sources of socioeconomic and health resource variables for Brazil, Ecuador and Mexico using open data sources from 2000 to 2019. We obtained 9142 municipalities from the three countries and used a method used in previous studies^16^ to obtain the best quality records. We selected 4894 municipalities that had adequate data quality according to five indicators for the 2000–2002 period; (1) mean relative deviation of birth rate; (2) ratio of reported to estimated live births; (3) age-standardized mortality rate; (4) mean relative deviation of mortality rate; and (5) proportion of deaths with undetermined causes (Chapter XVIII, ICD-10). The final dataset included 1067 municipalities in Mexico, 3669 in Brazil and 158 in Ecuador. For more details about the quality data process, please see the Tables S1, S2 and Fig. S1.

### Data sources

Health data were collected from the National Institute of Statistics and Censuses (INEC) of Ecuador, Information System of the Unified Health System (DATASUS) of Brazil and General Directorate of Health Information (DGIS) of Mexico. We obtained the socioeconomic variables from the National Institute of Statistics and Censuses (INEC) of Ecuador, the Institute of Geography and Statistics (IBGE) of Brazil and the National Council for the Evaluation of Social Development Policy (CONEVAL) and the National Institute of Statistics and Geography (INEGI) of Mexico.

### Analytical variables

The dependent variable was under-five child mortality rate (U5MR), calculated as the number of deaths of children under five years of age per 100,000 children between 0 and 4 years of age. The independent variables were (a) Gini inequality index, (b) illiteracy (prevalence of illiteracy among individuals older than 15 years), (c) poverty, (d) sewage (percentage of households with inadequate sewers), (e) access to piped water, (f) number of beds per 1000 population and (g) number of physicians per 1000 population. The variables were selected based on the availability of data at the municipal level in the three countries. All variables were obtained at the municipal level. The list of all data sources with references and detailed description of each variable, as well as the source and years obtained, can be found in Table S3.

The methodology applied for data collection, cleaning, interpolation, and analysis was standardized and consistent across the countries. For more details on the interpolation method please see the Figs. S2–S8. The analysis procedures, including the application of machine learning algorithms such as the random forest model, were identical across the three countries. This uniform approach facilitated a fair and direct comparison of results.

### Statistical and machine learning analysis

We conducted a descriptive analysis with all the variables considered, estimating the mean, standard deviation and relative change for all the years considered. We randomly divided the total data into 70% to train the models and 30% to obtain variable importance, cumulative dependency, and performance metrics. We trained into a random forest model^17^ as one of the best algorithms for predicting infantile mortality or under-five child mortality in other studies^12–14^. To train the random forest we used k-fold cross-validation technique with threefold.

We used an agnostic approach to find the most important variables to predict under five-mortality rate. The agnostic model does not assume specific structures in the prediction models, which allows comparing the importance of the explanatory variables between different models^18^. The main idea is to evaluate how much the performance of a model changes when a variable is removed. Thus, if the variable is important to predict the under-five child mortality rate, the performance of the models will worsen proportionally. For this method we used 50 perturbations (or resamples) with the training data to obtain the root mean square error (RSME; measures the amount of error between the data predicted by the model and the true data) missing from the total of 50 resamples for each variable. Subsequently, we obtained the cumulative dependence between the main predictors and the under-five child mortality rate. The cumulative dependency plots show the relationships found by the algorithms between a predictor and the dependent variable considering the correlations between the predictors.

Finally, to compare the prediction goodness of the three trained models, we calculated the four most used metrics^18^: the root-mean-squared-error (RSME), the mean squared error (MSE; can be viewed as a sum of squared residuals), the median absolute deviation (MAD; can be seen as a correction of the MSE to outliers) and ${r}^{2}$. We conducted two more additional analysis: (i) we trained random forest model for each country to capture possible biases in the measurement of the variables used between countries: (ii) we trained random forest model for all countries and for specific causes of under-five child mortality rate: (a) nutritional deficiencies (D50-D53, D64.9, E00-E02, E40-E46, E50-E64, ICD-10)^19^ and (b) due to respiratory infections (H65-H66, J00-J22, P23, U04, ICD-10)^19^. The results of these analysis are found in the Figs. S9–S13.

### Sensitivity analysis

We trained two different machine learning models: (a) gradient bootstrap machine (GBM)^20^ and (b) generalized additive model using splines (GAM)^21^ to verify the robustness of our results.

### Data availability

The data that support the findings of this study are available from the corresponding author upon reasonable request.

## Results

Table 1 shows results of the descriptive analysis form all countries over the complete period. Gini index, illiteracy and poverty rates decreased over the years (15.35%, 46.73% and 54.39%), respectively, for the entire period considered. However, access to water, sewage, and physicians’ rate showed an increased trend over the years. Finally, the under-five child mortality rate has decreased considerably from 450.7 deaths per 100,000 inhabitants in 2000 to 271.02 deaths per 100,000 inhabitants in 2019 (a 39.86% decrease).

**Table 1:** Descriptive analysis for all countries.

Variables	Year			Relavite change (%)
	2000	2010	2019	
Dependent variables				
Population 0–4 years	4869.75 (20,137.74) [72—924,023]	4416.89 (17,778.72) [32—789,227]	4425.46 (17,825.41) [39—792,794]	− 9.12
U5MR-N	450.7 (298.93) [0—3676]	297.68 (226.01) [0—3125]	271.02 (228.57) [0—2632]	− 39.86
Independent variables				
Sociodemographic				
Gini index	52.04 (7.68) [26.92—81]	46.21 (7.35) [26.65—80]	44.05 (9.66) [9.81—92.01]	− 15.35
Illiteracy rate	16.89 (10.76) [0.83—74.88]	12.44 (8.28) [0.56—58.68]	8.98 (6.84) [0—54.25]	− 46.73
Poverty	34.89 (19.71) [0.7—96.49]	21.95 (17.05) [0—94.86]	15.91 (17.08) [0—100]	− 54.39
Water	64.8 (23.14) [0—100]	73.71 (19.22) [0—100]	80.45 (18.89) [0—100]	24.15
Sewage	26.84 (25.23) [0—99.38]	40.54 (30.26) [0—100]	46.51 (32.53) [0—100]	73.28
Health resources				
Physician rate	0.63 (0.58) [0—6.57]	0.67 (0.61) [0—6.22]	0.89 (0.94) [0—29.79]	41.26
Hospital beds rate	2.08 (2.56) [0—46.43]	1.81 (2.13) [0—31.11]	1.66 (2.07) [0—30.2]	− 20.19

Average (standard deviation) [minimum—maximum].

**Figure 1:**
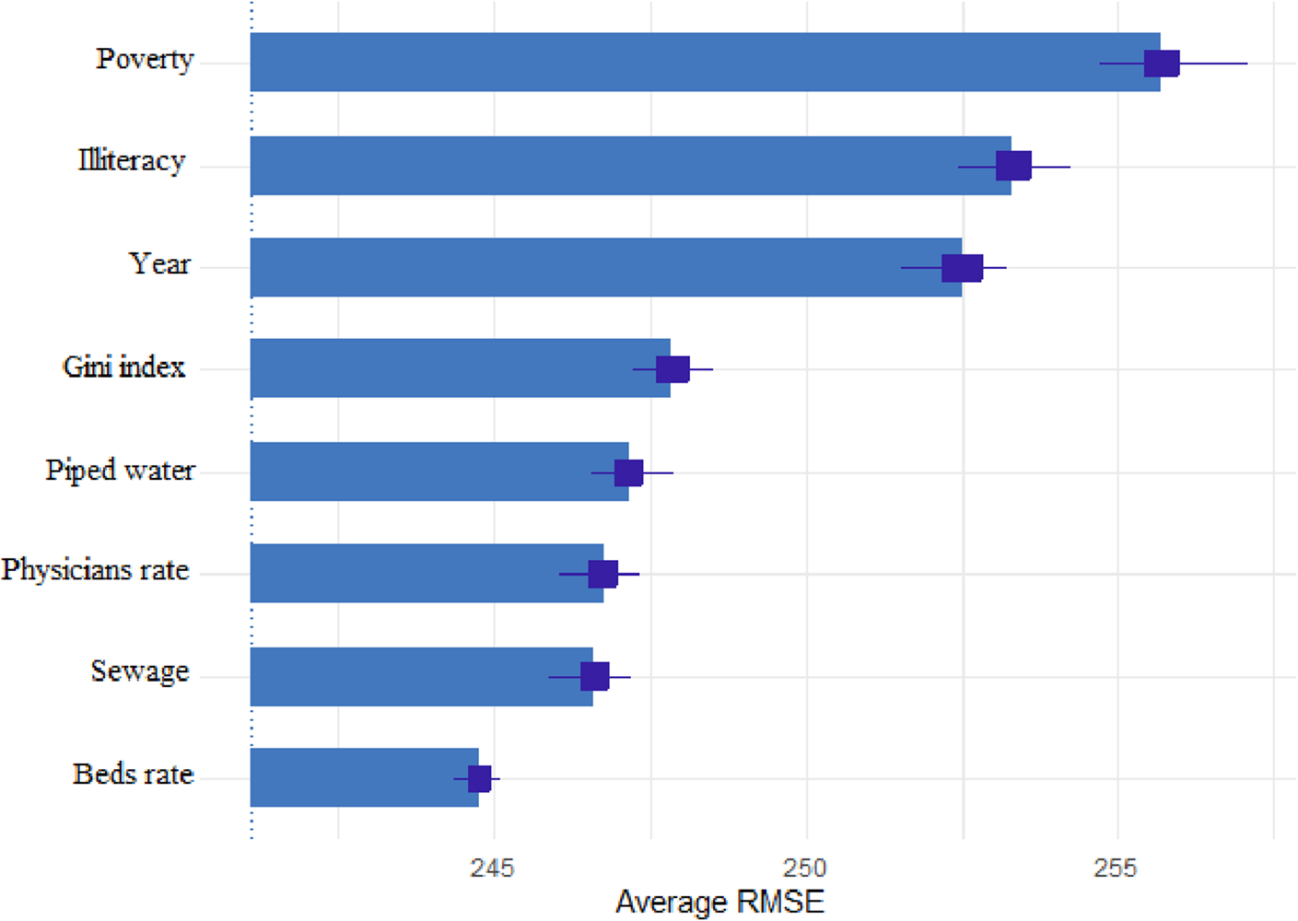
Importance of the variables for random forest algorithm. *Note*: The plot represents the average error to predict the U5MR gained by the model if each variable is removed. The greater the error gained, the more important the variable is for predicting U5MR.

Figure 1 shows the importance of different social determinants in predicting under-five child mortality rate for the random forest model, which was the best performing model (RMSE = 241.08, MSE = 58,120.88, MAD = 120.48, ${r}^{2}$= 0.16). The bar graphs represent the average RMSE lost when each variable is removed, while the box-and-whisker plot represents the distribution of RMSE lost for the 50 perturbations. For the random forest model all the variables considered were important in predicting under-five child mortality rate, however, poverty was the most important variable, followed by illiteracy, years, and the Gini index.

**Figure 2:**
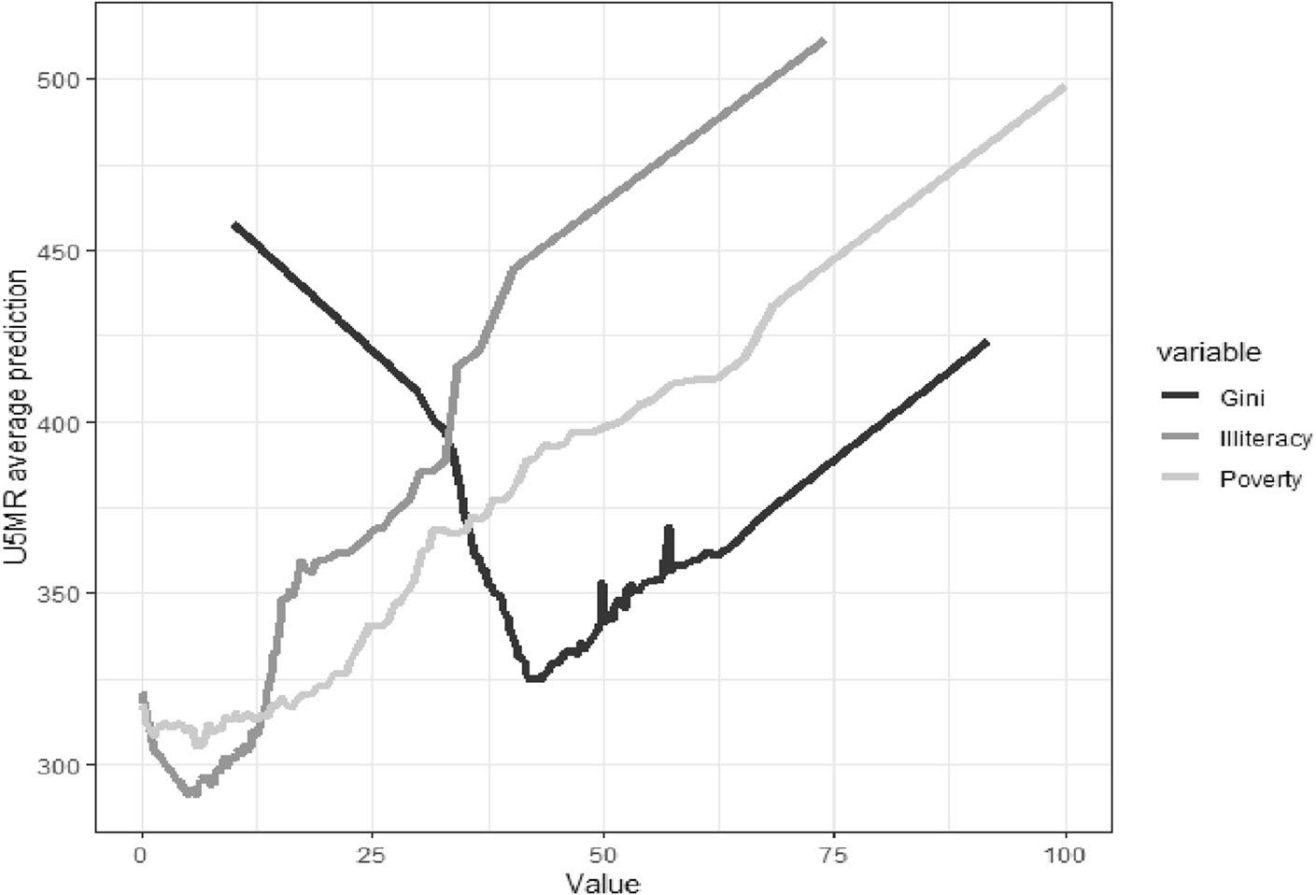
Cumulative dependency plot for random forest algorithm. *Note*: The graph represents the relationships found by the random forest algorithm between the three most important variables and the U5MR.

Figure 2 presents the cumulative dependency plots between the under-five child mortality rate and the three most important variables for the random forest algorithm. A positive relationship is observed between poverty and U5MR. At the same time, a positive and steeply sloping relationship between illiteracy and the under-five child mortality rate can be observed. Finally, a non-linear relationship can be observed between the Gini index and the average prediction of under-five child mortality rate where there is a u-shaped relationship between these two variables.

**Figure 3:**
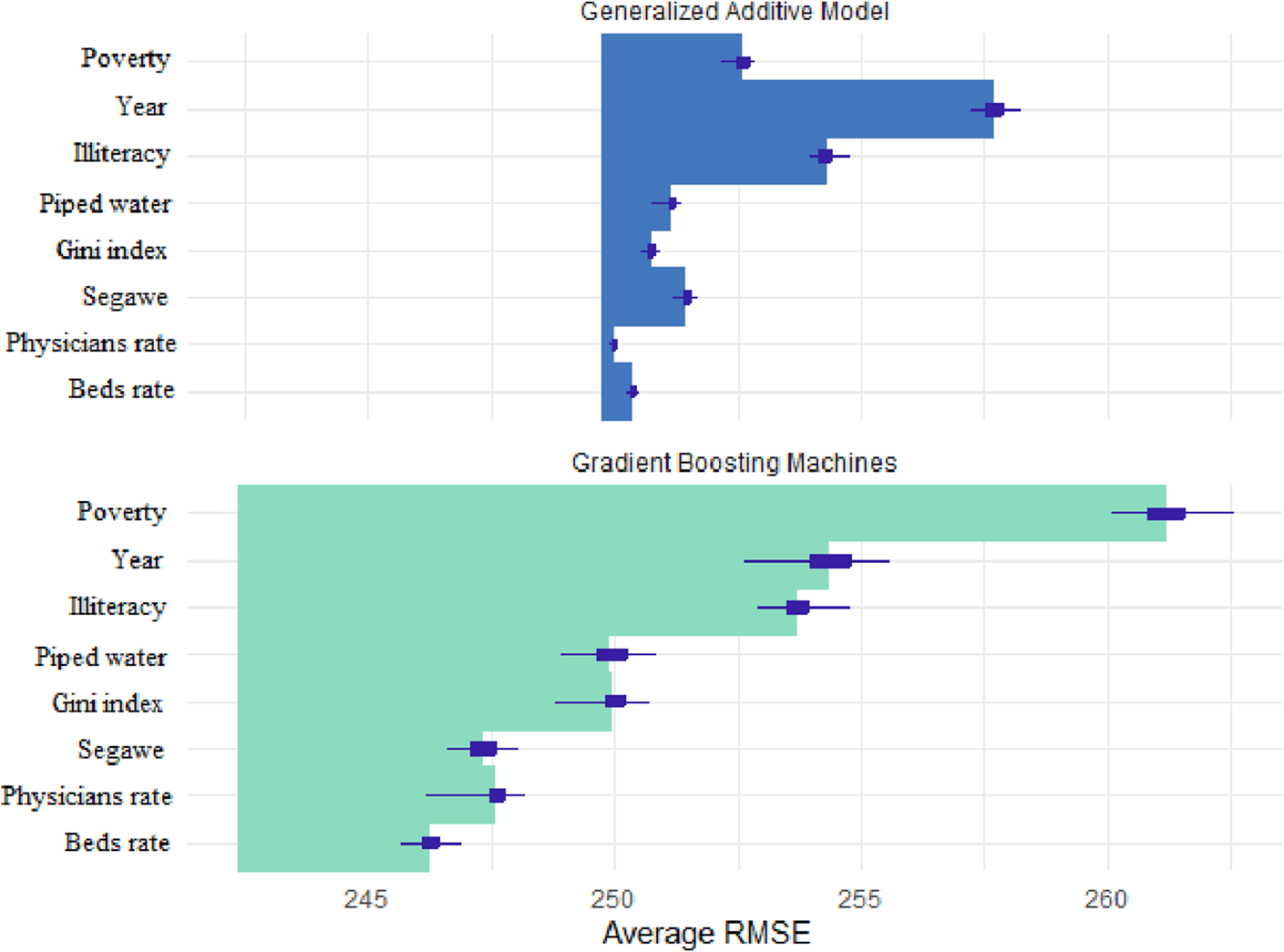
Importance of variables for GAM and GBM algorithms. *Note*: The plot represents the average error to predict the U5MR gained by the model if each variable is removed. The greater the error gained, the more important the variable is for predicting U5MR.

Figure 3 shows the results of sensitivity analysis. The importance of most of the variables for the two different algorithms was consistency. For both algorithms, poverty and illiteracy and were the most important variables in predicting under-five child mortality. As can be seen, the GBM (RMSE = 242.36, MSE = 58,741.78, MAD = 122.47, ${r}^{2}$= 0.15) performed better than the GAM (RMSE = 249.74, MSE = 62,373.82, MAD = 127.14, ${r}^{2}$= 0.1). As with the random forest algorithm, poverty and illiteracy were two important variables for predicting the under-five child mortality. Finally, for specific causes, the random forest model found that poverty, illiteracy and the Gini index were the most important social determinants of health for predicting mortality due to nutritional deficiencies. For deaths from respiratory infections random forest found that poverty, the Gini index, and sewage were the three most important. In both causes random forest models found that all variables were important in predicting mortality.

## Discussion

Our study addressed a central issue in the efforts of low- and middle-countries to decrease under-five child mortality. Taking the case of Brazil, Ecuador, and México over the last two decades as a reference, and combining innovative research methods such as machine learning, our findings provide original on the importance of each social determinants on child mortality, overall and for specific causes In this respect, five results stand out: (1) Compared to GBM and GAM, the random forest algorithm was the best performing prediction model; (2) Poverty, illiteracy, and the Gini index were the three most impactful social determinants associated with U5MR; (3) There was a positive relationship between poverty and illiteracy with 5UMR, and a u-shaped relationship between Gini index and 5UMR; (4) results from the sensitivity analysis were shown to be consistent, since poverty and illiteracy were also the most important social determinants to predict U5RM; (5) Poverty and illiteracy were the most important predictors of mortality from nutritional deficiencies and respiratory infections.

In this research, while the core focus revolved around exploring the social determinants of under-five child mortality in three Latin American countries, what sets our study apart is the method of analysis employed. The application of machine learning, particularly the Random Forest model, in the context of understanding the intricate relationships between social determinants and under-five child mortality represents a notable departure from traditional analytical approaches. The utilization of machine learning algorithms in a longitudinal dataset (up to 20 years and 3 different country) allows for the automatic identification of interactions and the identification of both linear and nonlinear relationships between the target variable (under-five child mortality) and the independent variables (selected social determinants). This aspect is particularly critical in comprehending the nuanced and complex interactions that contribute to under-five child mortality. In contrast, traditional linear models might overlook these nonlinear relationships. Additionally, compared to traditional statistical methods like linear regressions, the machine learning models we use don't require statistical assumptions. In most use of machine learning models in health, researchers face the dilemma of good model performance vs good interpretability^18^, however with the agnostic approach that we used we were able to select the best model to predict U5MR without sacrificing interpretively that is crucial in the health area. In our study in the three machine learning models, poverty was most important social determinants of health for predicting under-five child mortality in multiple studies different measures of poverty have shown that it is one of the most important social determinants of health to predict infant mortality or under-five child mortality. For example, Saroj et al. and Malderen et al.^12,22^ found that household wealth was one of the most important social determinants of health for predicting under-five child mortality in Africa. In turn, Bizzego et al.^13^ found that household wealth, household sanitation and availability of drinking water were associated with infant mortality. Using machine learning models, Hemo & Rayhan^15^ found that wealth index was one of the most important social determinants of health for predicting malnutrition problems in children.

We found that illiteracy was the second most important variable for predicting under-five child mortality rate for the two decades evaluated in the three countries considered. These results are consistent with multiple studies that have found relationships between educational level in the general population, in women or in parents at the individual, municipal or multi-country. For example, some studies^12,13,22,23^ found a negative relationship between under-five child mortality or infantile mortality and the educational level of women or mothers with survey data at the individual level. Hemo & Rayhan^15^ found a negative relationship between parental education level and malnutrition problems in children. In an ecological study, Mukherjee et al.^23^ found that female literacy was the variable that most negatively predicted under-five child mortality. In a multi-country study, Schell et al.^24^ found that female illiteracy was one of the variables that most explained the difference between countries on infant mortality with a positive relationship. These findings were presented for low-income countries where illiteracy was the most important variable, while for middle-income countries it was the Gini index that was most important. Similarly, Prisco et al.^25^ found a positive relationship between low female education and infant mortality in European Union countries. Our results add to a growing literature that points to educational social determinants of health as one of the most important or the most important social determinants of health for predicting under-five child mortality regardless of the level of analysis at which it is measured.

On the other hand, we found that the Gini index was the third most important social determinants of health for predicting under-five child mortality, however we found a non-linear relationship between these two variables. At low and medium levels of Gini index (from 0 to 0.45 approximately) the Gini and under-five child mortality are negatively related, however from medium and high levels of Gini (over 0.45 approximately) the relationship becomes positive. This result is consistent with other studies. For example, Schell et al.^24^ found that Gini index was among the three variables explaining infant mortality, however, these authors found that Gini was important mainly in middle-income countries. Siddiqi et al.^26^ found negative relationships between Gini and infant mortality, however when including the interaction between Gini and time they found that the association was positive. Siddiqi^26^ suggest that the effect of income inequality on infant mortality depends on time and its interaction with other social conditions. For their part, Lazariva and Prisco et al.^25,27^ found no relationship between the Gini index and infant mortality. In the same way, Avendano^28^ also found no effect of the Gini index on infant mortality when controlling for differences between countries. Avendano^28^ considers that social policies that reduce infant mortality are mainly concentrated in countries with low inequality, which may affect the possible effect of the Gini on infant mortality. More studies using non-linear models are needed to explore this relationship.

Our results showed that the random forest model had less error in predicting under-five child mortality. One of the problems of machine learning models is overfitting to the data, however, to avoid overfitting we trained the three machine learning models with a random sample of 70% and evaluated the performance, obtained the importance of the variables and the cumulative dependence with the remaining 30% of the data. Although machine learning models do not have hypothesis testing like classical linear models, several methods have been implemented to increase the interpretability of machine learning models^18^. We use the agnostic approach that allows multiple models to be interpreted regardless of their internal structure. We use the method of variable importance by perturbations. The main drawback of the permutation-based variable importance measure is its dependence on the selected subsamples. Consequently, for different permutations we will obtain, in general, different results. Nevertheless, we use 50 perturbations to minimize as much as possible the error derived from random sampling. Another possible limitation is that not all variables were measured in the three countries, specifically poverty was measured differently in each country, however we trained one model per country taking into account this limitation.

Our research did introduce a potential selection bias by excluding municipalities with lower data quality, it is essential to emphasize that this bias does not significantly impact the validity of our results. Despite this limitation, we found that derived from the data quality method that we applied, we only excluded 14% of the population of the three countries (taking 2000 as the reference year), so the vast majority of the population of the three countries was taken into account in our analysis. The rigorous data quality assessment process was aimed at ensuring the reliability and accuracy of the data used in our analysis, particularly for vital information. Although the excluded municipalities tended to have greater socio-economic disadvantages, we found that the variable most crucial to our study, poverty, did not significantly bias our results, in some case our results could be sub estimated. Moreover, the fact that other studies in Mexico have identified a relationship between poverty and high mortality rates further supports the consistency of our findings. The decision to prioritize data quality over quantity not only increased the internal validity of our study but also enhanced the overall robustness and reliability of our conclusions. We acknowledge the limitation of some external validity due to the selection bias, but we believe that the trade-off was justifiable, as the benefits of utilizing high-quality data far outweigh the potential drawbacks. Therefore, we remain confident that our study's results provide meaningful and accurate insights into the municipalities with higher data quality in Brazil, Ecuador, and Mexico.

## Conclusions

Our findings have important implications for Latin America and all low- and middle-income countries. Despite the fact that we have observed an increase in the availability of human and material resources for health, sewage and piped water services, we have also observed a decrease in poverty, illiteracy, and inequality.

Child mortality continues to be a problem that mainly affects developing countries such as those in Latin America and Caribbean. Slowing economic growth, weak employment, high inflation rates are deepening and prolonging the economic and social crisis in Latin America and Caribbean. According to CEPAL^29^, extreme poverty in Latin America and Caribbean went from 13.1% in 2020 to 13.8% in 2021, representing a reversal of approximately 27 years. Poverty will reach 32.1% of the total population in Latin America and Caribbean by the end of 2022. At the same time, inequality has also been increasing in recent years in this region^29^. In addition, the impact of the pandemic caused by COVID-19 has also affected the education sector in this region, mainly due to the prolonged interruption of face-to-face education, which may sharply increase illiteracy rates that had been reduced for years. In this same context, according to the Development Bank of Latin America^30^, the number of vulnerable people has also grown in recent years in Latin America and Caribbean. Vulnerable people are those who are at high risk of falling into poverty due to an event such as a temporary loss of income. According to this organization, the number of vulnerable people increased from 37% in 2019 to 38.5% in 2022. These people are not usually covered by conditional transfer or basic social protection programs, which further increases their probability of falling into poverty aligning this panorama with the findings in this study, it is mandatory to formulate public policies focused on improving people's living conditions in terms of education, housing, income equity, access to health, among others, they would be a fundamental tool in reducing infant mortality and guaranteeing the right to children's life.

Our results show that it is important to analyze child mortality from a multidimensional perspective, where socioeconomic, educational and specific approaches to the Health System of each country are contemplated. The results of our analyzes make explicit the relationship that exists between the inequality of socioeconomic backgrounds with child mortality. Thus, an approach that extends beyond the limits of health, where a multisectoral public policy approach is needed, capable of acting not only on health organizations, but also on social and economic aspects, prioritizing populations that suffer deeper levels of social vulnerability. Moreover, the distribution of government resources must be more equitable in order to have effective public policies in health care, in essence, a mechanism for the redistribution of social advantages and disadvantages. According to the results, prioritizing the allocation of public policies in the populations with the greatest poverty and the lowest educational level could have a greater effect in reducing infant mortality.

### Supplementary Information

Supplementary Information.

## Data Availability

Health data were collected from the National Institute of Statistics and Censuses (INEC) of Ecuador, Information System of the Unified Health System (DATASUS) of Brazil and General Directorate of Health Information (DGIS) of Mexico. The socioeconomic variables were collected from the National Institute of Statistics and Censuses (INEC) of Ecuador, the Institute of Geography and Statistics (IBGE) of Brazil and the National Council for the Evaluation of Social Development Policy (CONEVAL) and the National Institute of Statistics and Geography (INEGI) of Mexico. The datasets used and/or analyzed during the current study are available from the corresponding author on reasonable request. All the data sources are provided in the “Supplementary materials”.
