# Supplementary material for: Understanding the social determinants of child mortality in Latin America over the last two decades: a machine learning approach
Source: Sci Rep. 2023 Nov 27;13:20839. doi: 10.1038/s41598-023-47994-w (PMC10682478; doi:10.1038/s41598-023-47994-w)
Supplement: Supplementary file 1 — Supplementary Information. [file 41598_2023_47994_MOESM1_ESM.docx]

**Supplementary materials**

## **Quality of Vital Statistics Methods**

The civil registration and vital statistics (CRVS), despite of being an important public health instrument for planning and evaluation, in most of the developing countries have still low quality and coverage [1]. In that regard, a study [2] involving 148 countries shows that the most of places with medium to low quality of CRVS during the period 1980-2012 were in the African, Asian Latin America regions, which reinforces the necessary care and attention of studies that work with CRVS in these regions. However, countries have been encouraged to stimulated by the need to monitor progress and accountability of CRVS, especially since 2015 for the Sustainable Development Goals (SDG) [1].

From a methodological point of view, the good quality of these CRVS is also crucial to guarantee the veracity of the results, especially in impact evaluation studies. Since our study uses municipal-level child death and livebirth data in Latin America and Caribbean countries, it is essential to separate those municipalities with high and low quality from these CRVS.

To mitigate this, we followed a methodology [3] to calculate the level of quality of CRVS of municipalities, which has been widely used in previous studies in Latin America and Caribbean countries [4-7]. This methodology used a validated multidimensional criterion based on five indicators: (1st) relative mean deviation of the birth-rate; (2nd) ratio of reported-to-estimated livebirths; (3rd) age-standardized mortality rate; (4th) relative mean deviation of the mortality rate; and (5th) proportion of deaths with undetermined causes (Chapter XVIII, ICD-10) [3].

After calculating these indicators, we made a weighted average of them to obtain a final indicator, followed by the division by terciles of the distribution of each country separately, so that the municipalities within the last two terciles were considered to have good quality of CRVS, and the first tercile refer to municipalities with low quality of CRVS.

As the quality CRVS tends to improve in more recent periods, we chose to apply this methodology in the period from 2000 to 2002, as it is the beginning of the historical series worked on in this paper and, at the same time, captures the beginning of the implementation of CCT programs.

After applying this method to all 7,204 municipalities in BEM with data available in 2000-19 period, we selected a subset of 4,894 municipalities that had the adequate quality of CRVS, which covered 68% of them, but representing 86% of the more than 340 million inhabitants of these 3 countries in 2002.

Additionally, the application of this method showed important socio-spatial inequalities: In Brazil, the proportion of adequate vital statistics was higher in the Center-South of the country and in the larger municipalities, and lower in many municipalities in the North of the country (Amazon region) and some municipalities in the Northeast. In Ecuador, the lowest results are located in the eastern region of these countries, especially in the Amazon region, while the municipalities with the highest QVI are from Andean regions. In Mexico, the best results are in the northern region of the country and the worst are in the southern region. The Table S1 and Figure S1 show these results.

**Table S1. Number of municipalities and population before and after filter by adequate CRVS.**

| Country | Number of municipalities | | | | By population | | |
| --- | --- | --- | --- | --- | --- | --- | --- |
|  | With data available during 2000-19 period | Filter by adequate quality of CRVS (2000-02) | % of municipalities with adequate quality of the total | Total | | Filter by adequate quality of CRVS (2000-02) | % of population with adequate quality of the total |
| Brazil | 5,507 | 3,669 | 67% | 178,135,381 | | 155,200,666 | 87% |
| Ecuador | 224 | 158 | 65% | 13,232,884 | | 11,202,804 | 85% |
| Mexico | 1,473 | 1,067 | 72% | 110,576,814 | | 95,918,270 | 87% |
| BCEM | 7,204 | 4,894 | 68% | 301,945,079 | | 262,321,740 | 86% |

Source: Author’s analysis of data from 2000-02 from SIM (DATASUS – Brazil), INEC (Ecuador), and INEGI (Mexico).

**Figure S1. Municipalities according to the quality of vital information**

| BRAZIL | ECUADOR |
| --- | --- |
| 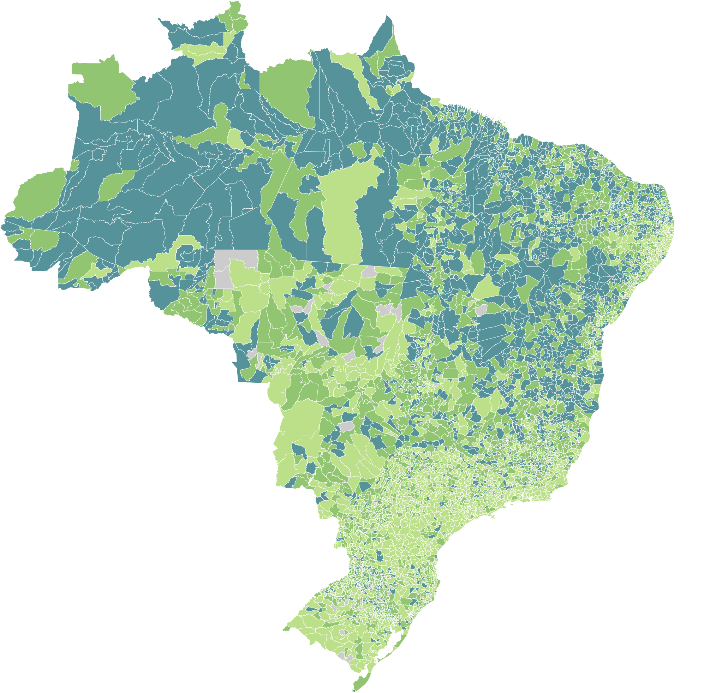 | 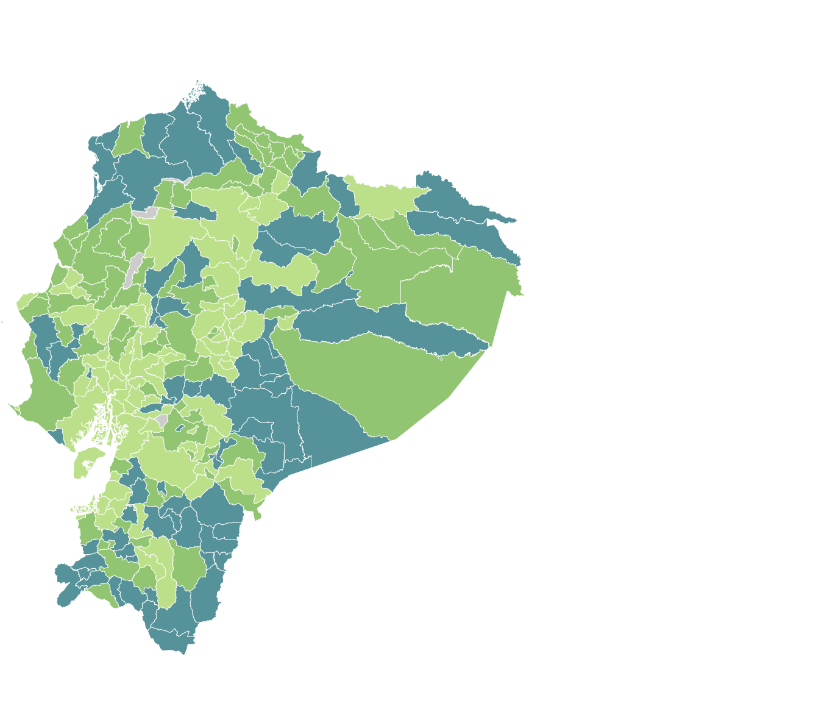 |
| MEXICO | BEM COUNTRIES |
| 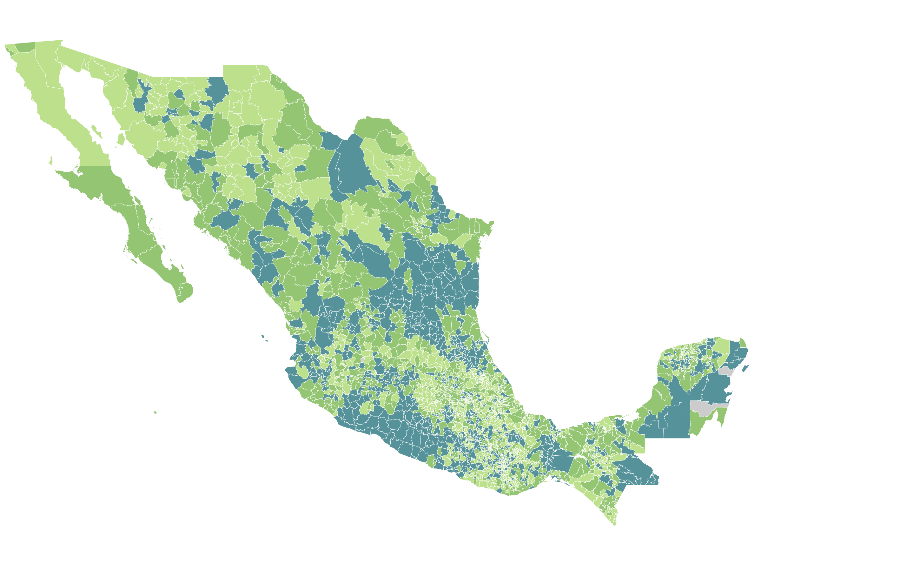 | 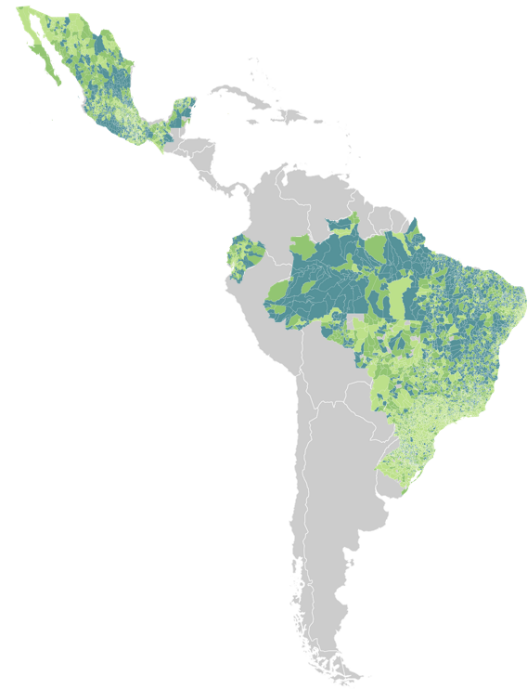 |

Low quality of CRVS Good quality of CRVS High quality of CRVS Municipalities created after 2001 or other countries not contemplated in this study

**Source**: Author’s analysis of data from 2000-02 from SIM (DATASUS – Brazil), INEC (Ecuador), and INEGI (Mexico).

Additionally, we obtained descriptive statistics of social determinants used to compare them between those municipalities considered and those not considered. The results are shown in table S2.

**Table S2. Tests of differences between independent variables between municipalities excluded and included.**

| Independ variable | Included municipalities | Excluded municipalities |  |
| --- | --- | --- | --- |
|  | Mean (SD) | Mean (SD) | Kolmogorov-Smirnov test |
| Gini inequality index | 46.91 (8.16) | 49.85 (8.77) | <.001 |
| Illiteracy | 12.75 (8.99) | 18.62 (10.11) | <.001 |
| Poverty | 22.78 (19.25) | 34.74 (20.54) | <.001 |
| Sewage | 37.53 (30.45) | 55.35 (28.51) | <.001 |
| Access to piped water | 73.66 (20.68) | 63.22 (24) | <.001 |
| Number of beds per 1,000 population | 1.84 (2.22) | 1.3 (1.78) | <.001 |
| Number of physicians per 1,000 population | .69 (.66) | .43 (.38) | <.001 |
| Number of municipalities | 4,894 | 2,310 |  |

| **Data source**The data used in this study were obtained from various governmental platforms. All the variables used in this study are aggregated to the municipal level. However, the data for some variables were not available for specific years and municipalities, therefore we performed an exponential decay method for interpolation, as detailed in Interpolation and extrapolation method section of this supplementary document. **Table S3. Variables, description, and years available and source for each country.** | | | | |
| --- | --- | --- | --- | --- |
| Country | Variables | Description | Original data Availability (years) | Source |
| Brazil | Gini | Gini Index | 2000 and 2010 = Census, at municipality level. From 2001 to 2012 = PNAD, at state level. From 2013 to 2019 = PNADC, at state level | IBGE - Census; IBGE - PNAD; and IBGE - PNADC |
|  | Illiteracy rate | Perc. of indiv. older than 15 years who are illiterate | 2000 and 2010 = Census, at municipality level. From 2001 to 2012 = PNAD, at state level. From 2013 to 2019 = PNADC, at state level | IBGE - Census; IBGE - PNAD; and IBGE - PNADC |
|  | Poverty | Poverty Rate | 2000 and 2010 = Census, at municipality level. From 2001 to 2012 = PNAD, at state level. From 2013 to 2019 = PNADC, at state level | IBGE - Census; IBGE - PNAD; and IBGE - PNADC |
|  | Piped water | Perc. of hh with piped water | 2000 and 2010 = Census, at municipality level. From 2001 to 2012 = PNAD, at state level. From 2013 to 2019 = PNADC, at state level | IBGE - Census; IBGE - PNAD; and IBGE - PNADC |
|  | Sewage | Perc. of hh with sewage/sanitation | 2000 and 2010 = Census, at municipality level. From 2001 to 2012 = PNAD, at state level. From 2013 to 2019 = PNADC, at state level | IBGE - Census; IBGE - PNAD; and IBGE - PNADC |
|  | Hospital beds rate | Hospital Bed Rate per 1,000 inhabitants without health insurance | 2000 to 2019 | DATASUS - CNES |
|  | Doctors rate | Number of Doctors per 1,000 inhabitants without health insurance | 2000 to 2019 | DATASUS - CNES |
|  | Population between 0 and 4 years | Number of population under 5 years | 2000 to 2019 | IBGE – Municipal population estimates |
|  | Mortality between 0 and 4 years | Number of death under 5 years | 2000 to 2019 | DATASUS - SIM |
| Ecuador | Gini | Gini Index | 2001 and 2010 = Census, at municipality level. | INEC |
|  | Illiteracy rate | Perc. of indiv. older than 15 years who are illiterate | 2001 and 2010 = Census, at municipality level. | INEC |
|  | Poverty | Poverty Rate (Consumption poverty rate) | 2001 and 2010 = Census, at municipality level. | INEC |
|  | Piped water | Perc. of hh with piped water | 2001 and 2010 = Census, at municipality level. | INEC |
|  | Sewage | Perc. of hh with sewage/sanitation | 2001 and 2010 = Census, at municipality level. | INEC |
|  | Hospital beds rate | Hospital Bed Rate per 1,000 inhabitants without health insurance | 2000 to 2019 | INEC |
|  | Doctors rate | Number of Doctors per 1,000 inhabitants without health insurance | 2000 to 2019 | INEC |
|  | Population between 0 and 4 years | Number of population under 5 years | 2001 and 2010 = Census, at municipality level. | INEC |
|  | Mortality between 0 and 4 years | Number of death under 5 years | 2000 to 2019 | INEC |
| Mexico | Gini | Gini Index | 2000, 2005, 2010, 2020 | CONEVAL |
|  | Illiteracy rate | Perc. of indiv. older than 15 years who are illiterate | 2000, 2005, 2010, 2015, 2020 | CONEVAL |
|  | Poverty | Poverty Rate (Marginalization index) | 2000, 2005, 2010, 2020 | CONAPO |
|  | Water | Perc. of hh with piped water | 2000, 2005, 2010, 2015, 2020 | CONEVAL |
|  | Sewage | Perc. of hh with sewage/sanitation | 2000, 2005, 2010, 2015, 2020 | CONEVAL |
|  | Hospital beds rate | Hospital Bed Rate per 1,000 inhabitants without health insurance | 2001-2020 | Datos abiertos DGIS |
|  | Doctors rate | Number of Doctorss per 1,000 inhabitants without health insurance | 2001-2020 | Datos abiertos DGIS |
|  | Population between 0 and 4 years | Number of population under 5 years | 2000-2020 | CONAPO |
|  | Mortality between 0 and 4 years | Number of death under 5 years | 2000-2020 | INEGI |

## **Interpolation and extrapolation method**

Some of control variables are not available in total period of 2000-19. In these cases, we use the exponential decay method to extrapolate the variables available at least two points of time, and we drop municipalities with only one information. A total of 893,214 values were generated for 7 control variables, in 7,204 municipalities over 20 years with this method. The interpolated variables were Illiteracy rate, household infrastructure (sewage and piped water), hospital bed rate, doctor rate and inequality and income variables (Gini index and poverty rate). Some of these variables were used as control variables for the models, but none of the outcome variables (mortality and population) were interpolated. In the end, it was observed that the interpolated/extrapolated variables improved the control and precision of the models. The results of the interpolation method are seen in Figure S2-S8.

**Figure S2. Gini index boxplot for selected municipalities in Brazil, Ecuador and Mexico, BEM, from the period 2000-19.**

| EACH COUNTRY – GINI INDEX | ALL COUNTRIES (BEM) – GINI INDEX |
| --- | --- |
| 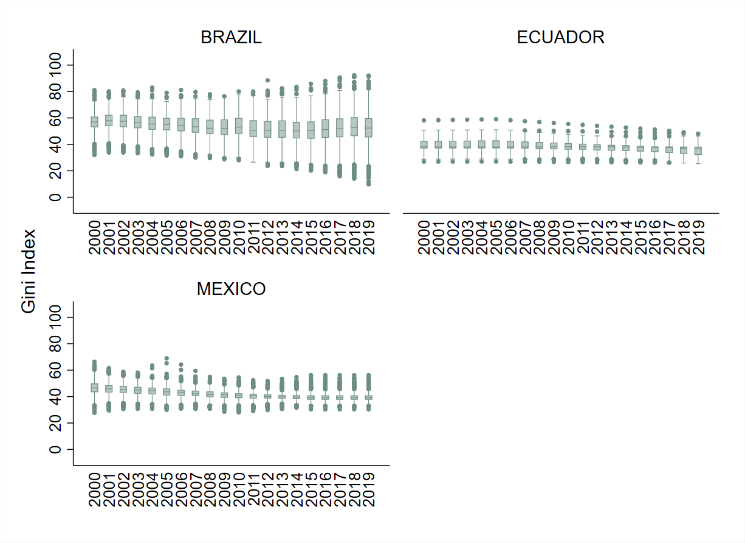 | 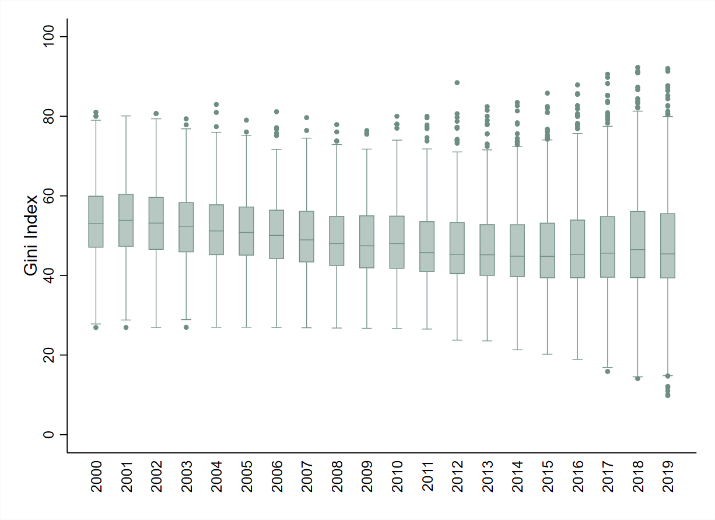 |

Source: Author's plot for 4,894 selected municipalities in Brazil, Ecuador, and Mexico, over 20 years (from 2000 to 2019).

Note: We selected municipalities with adequate quality of civil registration and vital statistics (CRVS). In Brazil, the gini index was available at the municipal level for the years 2000 and 2010, and the period 2001-09 and 2011-19 were extrapolated. Ecuador has the gini index available at the municipal level for the years 2005 and 2014, and we extrapolate the period 2000-04, and 2015-19. In Mexico, the gini index at the municipal level is available for the years 2000, 2002, and 2004-19, and we only extrapolate the years 2001 and 2003.

**Figure S3. Illiteracy rate boxplot for selected municipalities in Brazil, Ecuador and Mexico, BEM, from the period 2000-19.**

| EACH COUNTRY – ILLITERACY RATE | ALL COUNTRIES (BEM) – ILLITERACY RATE |
| --- | --- |
| 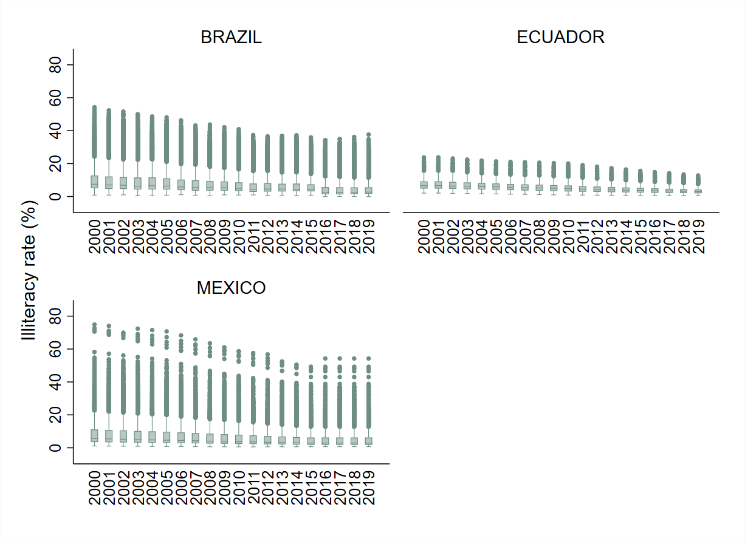 | 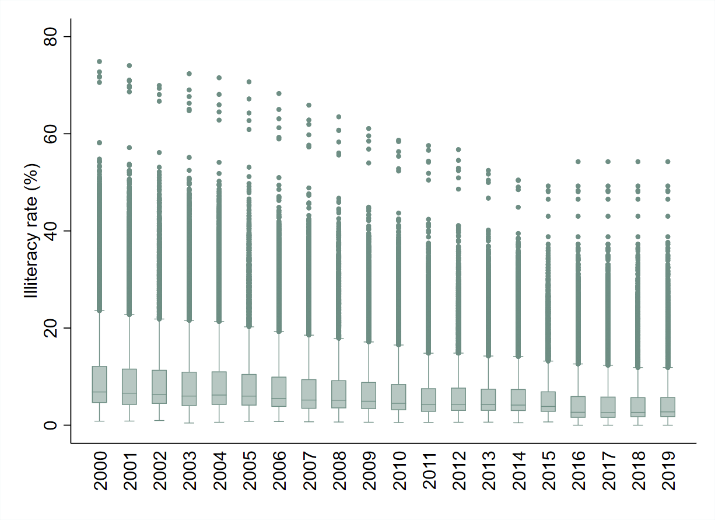 |

Source: Author's plot for 4,884 selected municipalities in Brazil, Ecuador, and Mexico, over 20 years (from 2000 to 2019).

Note: Illiteracy rate is the proportion of individuals older than 15 years who are illiterate. We selected municipalities with adequate quality of civil registration and vital statistics (CRVS). In Brazil, the illiteracy rate was available at the municipal level for the years 2000 and 2010, and the period 2001-09 and 2011-119 were extrapolated. Ecuador has the illiteracy rate available at the municipal level for the years 2001 and 2010, and we extrapolate the period 2002-09, 2011-19, and the year 2000. In Mexico, the gini index at the municipal level is available for the years 2000, 2005, 2010, 2015 and 2020, so we extrapolate the years 2001-04, 2006-09, 2011-14, and 2015-19.

**Figure S4. Poverty rate boxplot for selected municipalities in Brazil, Ecuador and Mexico, BEM, from the period 2000-19.**

| EACH COUNTRY – POVERTY RATE | ALL COUNTRIES (BEM) – POVERTY RATE |
| --- | --- |
| 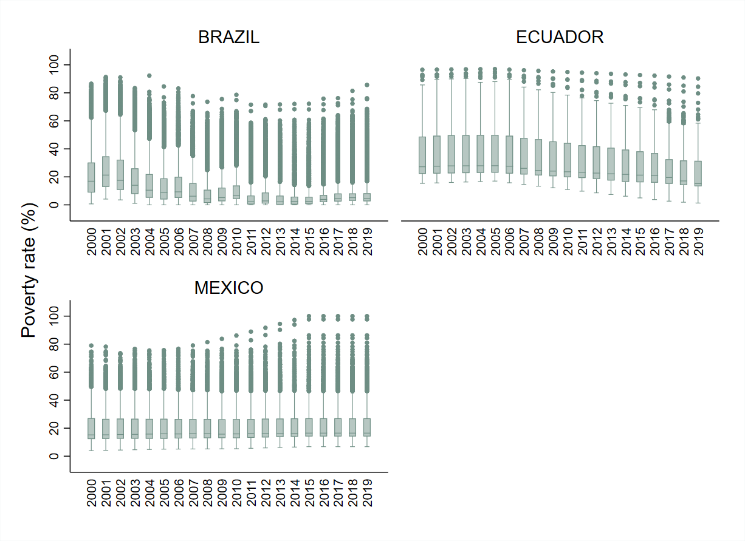 | 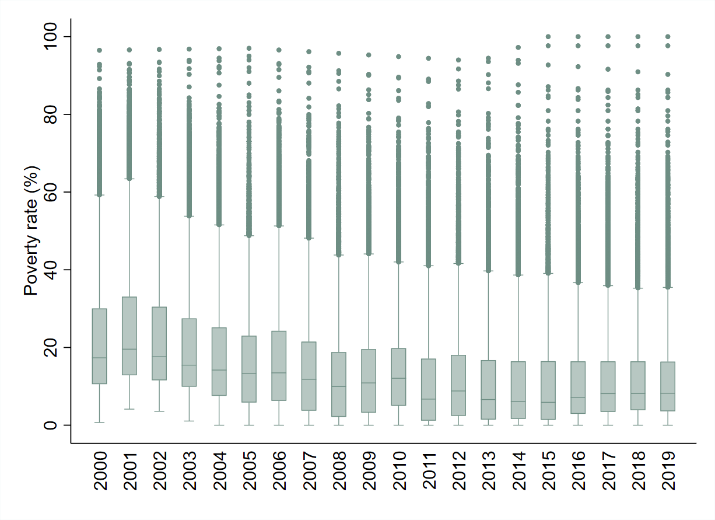 |

Source: Author's plot for 4,884 selected municipalities in Brazil, Ecuador, and Mexico, over 20 years (from 2000 to 2019).

Note: We selected municipalities with adequate quality of civil registration and vital statistics (CRVS). In Brazil, the poverty rate was available at the municipal level for the years 2000 and 2010, and the period 2001-09 and 2011-19 were extrapolated with microdata at state level. Ecuador has the poverty rate available at the municipal level for the years 2001 and 2010, and we extrapolate the period 2002-09, 2011-19, and the year 2000. In Mexico, the poverty rate at the municipal level is available for the years 2000, 2005, 2010, 2015 and 2020, so we extrapolate the years 2001-04, 2006-09, 2011-14, and 2015-19.

**Figure S5. Piped water boxplot for selected municipalities in Brazil, Ecuador and Mexico, BEM, from the period 2000-19.**

| EACH COUNTRY – PIPED WATER | ALL COUNTRIES (BEM) – PIPED WATER |
| --- | --- |
| 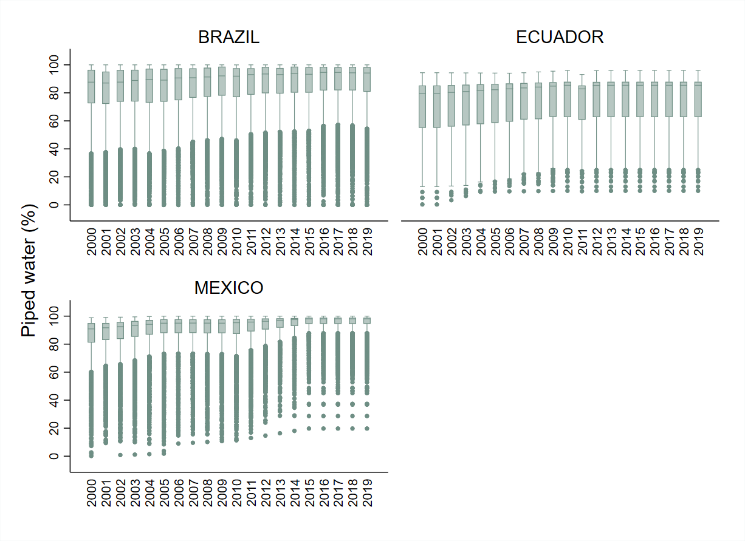 | 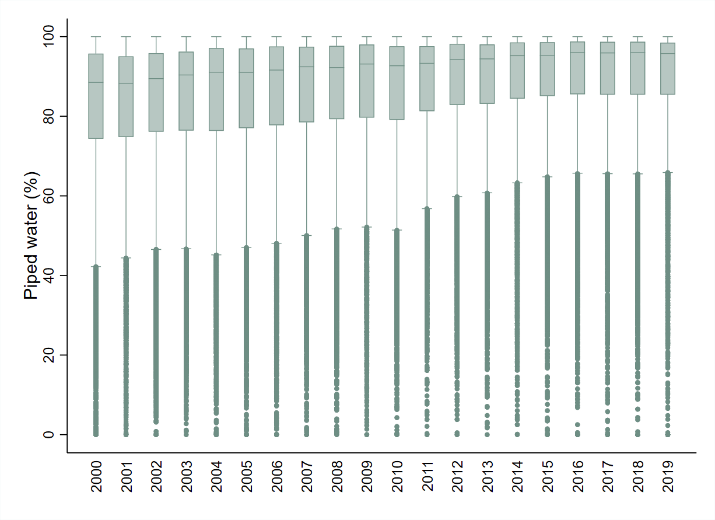 |

Source: Author's plot for 4,884 selected municipalities in Brazil, Ecuador, and Mexico, over 20 years (from 2000 to 2019).

Note: This variable refers is the proportion of individuals living in households with piped water. We selected municipalities with adequate quality of civil registration and vital statistics (CRVS). In Brazil, the piped water was available at the municipal level for the years 2000 and 2010, and the period 2001-09 and 2011-119 were extrapolated. Ecuador has the piped water coverage available at the municipal level for the years 2001 and 2010, and we extrapolate the period 2002-09, 2011-19, and the year 2000. In Mexico, the piped water at the municipal level is available for the years 2000, 2005, 2010, 2015 and 2020, so we extrapolate the years 2001-04, 2006-09, 2011-14, and 2015-19.

**Figure S6. Sewage boxplot for selected municipalities in Brazil, Ecuador and Mexico, BEM, from the period 2000-19.**

| EACH COUNTRY – SEWAGE | ALL COUNTRIES (BEM) – SEWAGE |
| --- | --- |
| 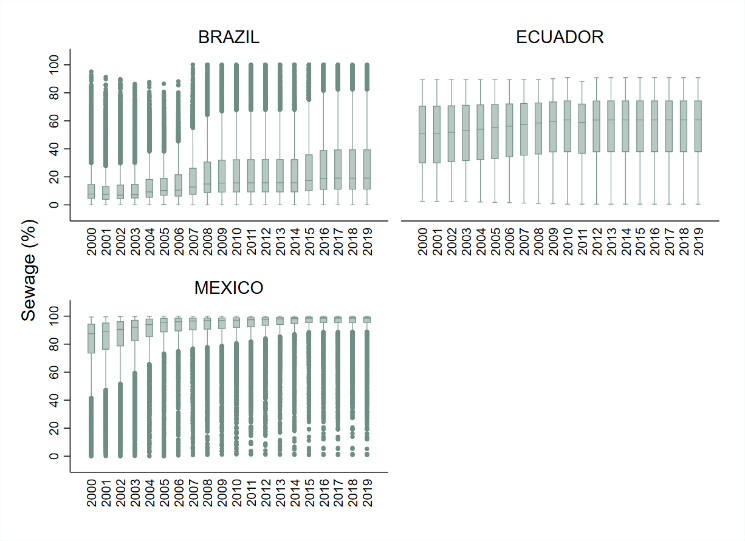 | 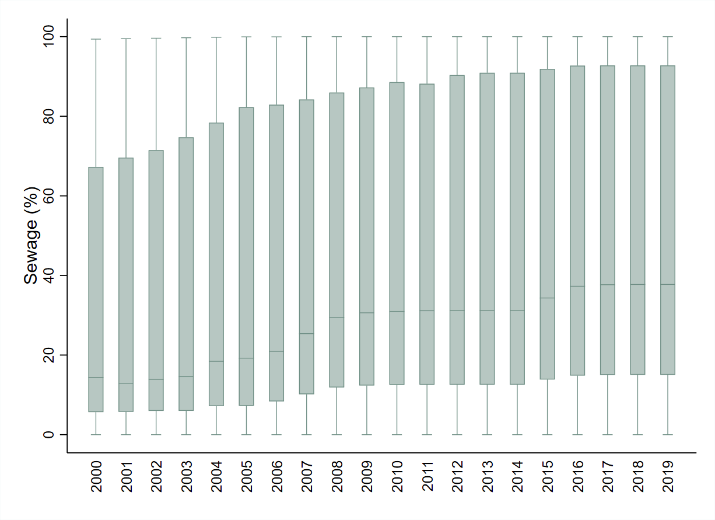 |

Source: Author's plot for 4,884 selected municipalities in Brazil, Ecuador, and Mexico, over 20 years (from 2000 to 2019).

Note: This variable refers is the proportion of individuals living in households with adequate sanitation. We selected municipalities with adequate quality of civil registration and vital statistics (CRVS). In Brazil, the sewage coverage was available at the municipal level for the years 2000 and 2010, and the period 2001-09 and 2011-19 were extrapolated. Ecuador has the sewage coverage available at the municipal level for the years 2001 and 2010, and we extrapolate the period 2002-09, 2011-19, and the year 2000. In Mexico, the sewage at the municipal level is available for the years 2000, 2005, 2010, 2015 and 2020, so we extrapolate the years 2001-04, 2006-09, 2011-14, and 2015-19.

**Figure S7. Physicians rate boxplot for selected municipalities in Brazil, Ecuador and Mexico, BEM, from the period 2000-19.**

| EACH COUNTRY – PHYSICIANS RATE | ALL COUNTRIES (BEM) – PHYSICIANS RATE |
| --- | --- |
| 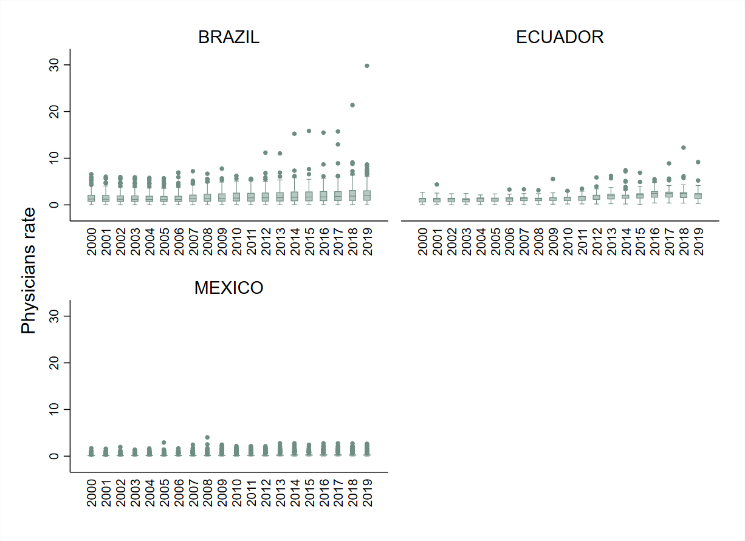 | 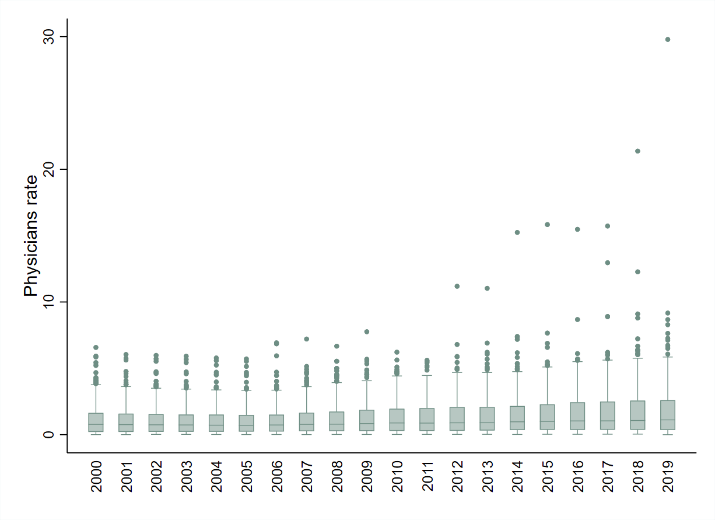 |

Source: Author's plot for 4,884 selected municipalities in Brazil, Ecuador, and Mexico, over 20 years (from 2000 to 2019).

Note: This variable refers to number of physicians per 1,000 inhabitants. We selected municipalities with adequate quality of civil registration and vital statistics (CRVS). In Brazil and Mexico, the physicians rate was available at the municipal level for the years 2000-19 (no need extrapolations). Ecuador has the physicians rate available at the municipal level for the years 2000-17, and we extrapolate the period 2018-19.

**Figure S8. Hospital beds rate boxplot for selected municipalities in Brazil, Ecuador and Mexico, BEM, from the period 2000-19.**

| EACH COUNTRY – HOSPITAL BEDS RATE | ALL COUNTRIES (BEM) – HOSPITAL BEDS RATE |
| --- | --- |
| 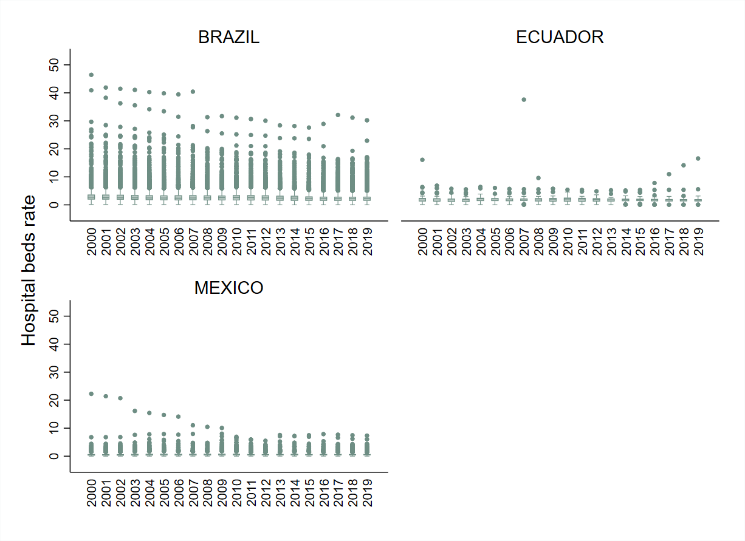 | 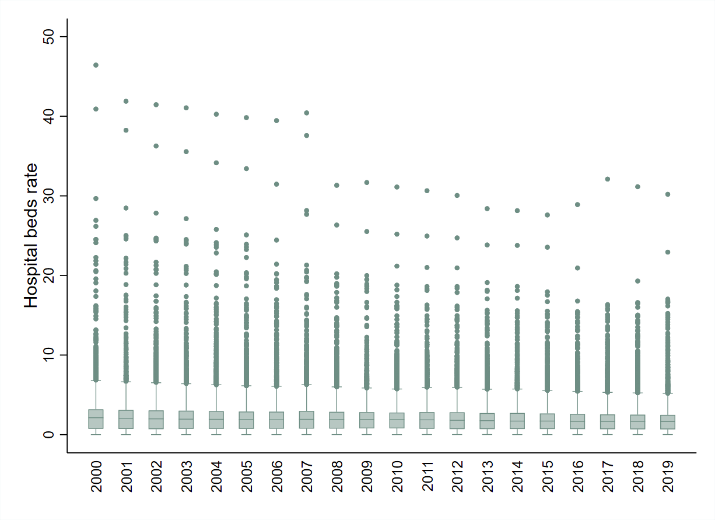 |

Source: Author's plot for 4,884 selected municipalities in Brazil, Ecuador, and Mexico, over 20 years (from 2000 to 2019).

Note: This variable refers to number of hospital beds per 1,000 inhabitants. We selected municipalities with adequate quality of civil registration and vital statistics (CRVS). In Brazil and Mexico, the hospital bed rate was available at the municipal level for the years 2000-19 (no need extrapolations). Ecuador has the hospital beds rate available at the municipal level for the years 2004-19, and we extrapolate the period 2000-03.

## **Machine learning results**

As complementary analyses, we introduced a Random Forest model for each country using the same agnostic approach that we used for the general results of our study (Figure S12-S16 show these results). Additionally, we introduced random forest models and obtained the importance of social determinants to predict the U5MR derived from nutritional deficiencies and infectious respiratory diseases for all countries considered (Figure S15 and S16 show these results).

**Figure S9. Importance of the variables to predict U5MR for Brazil.**


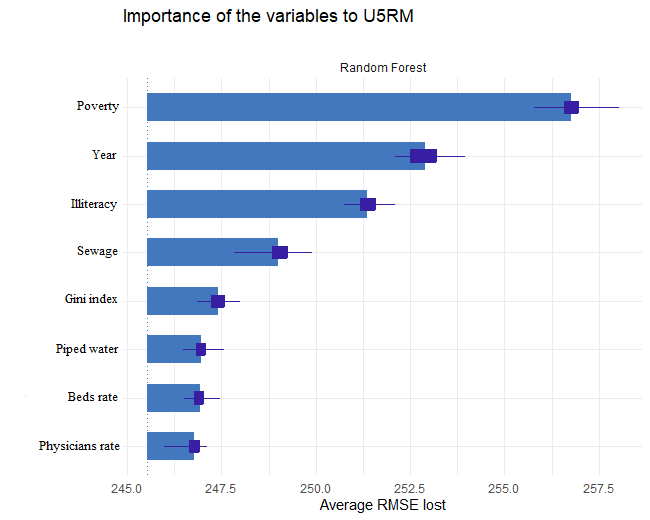


**Figure S10. Importance of the variables to predict U5MR for Ecuador.**


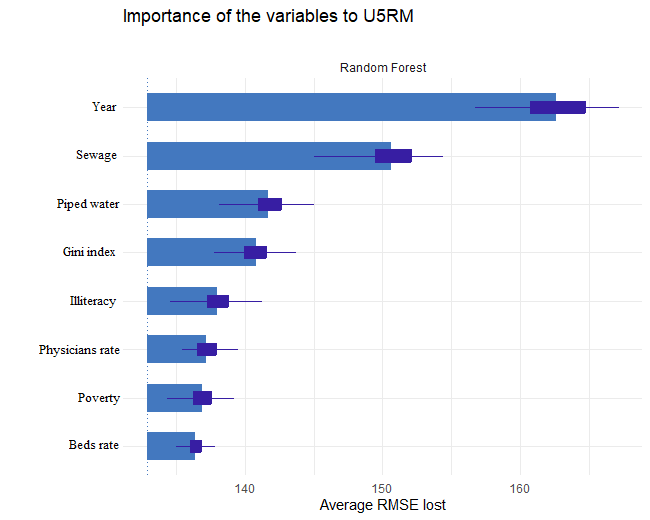


**Figure S11. Importance of the variables to predict U5MR for Mexico.**


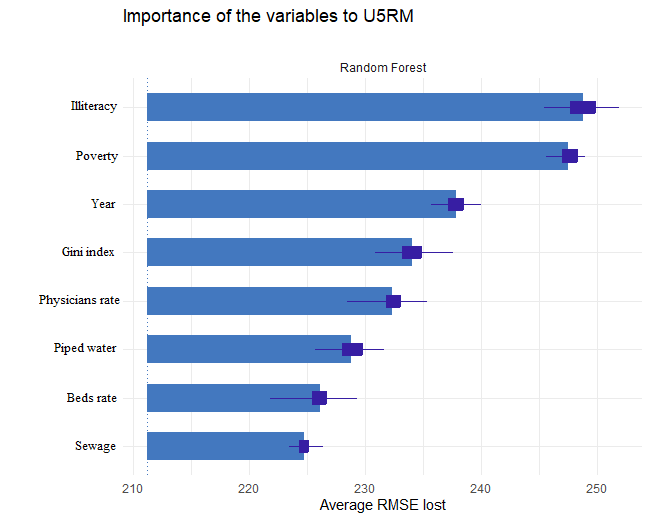


**Figure S12. Importance of the variables to predict U5MR due to nutritional deficiencies.**


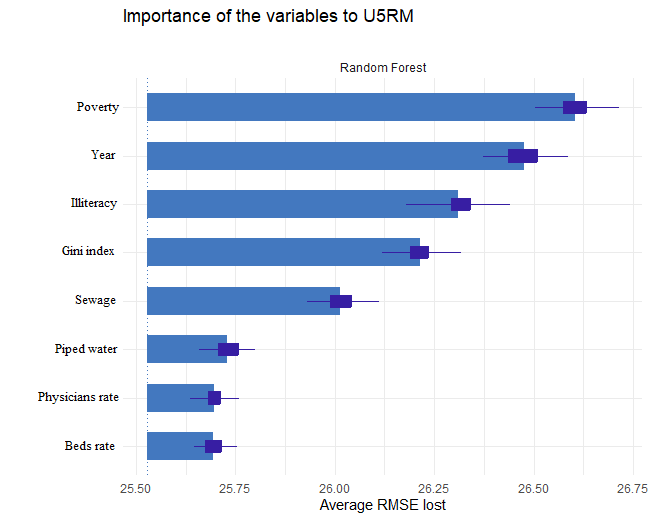


**Figure S13. Importance of the variables to predict U5MR due to respiratory infections.**


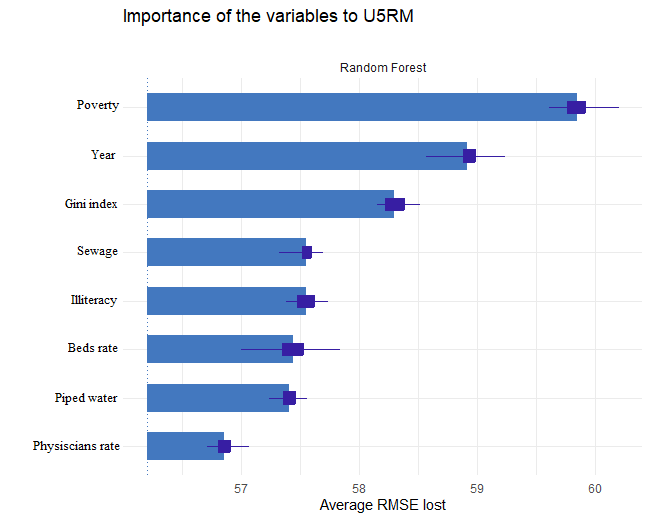


REFERENCES

[1] C. AbouZahr et al., “Civil registration and vital statistics: progress in the data revolution for counting and accountability,” The Lancet, vol. 386, no. 10001, pp. 1373–1385, Oct. 2015, doi: 10.1016/S0140-6736(15)60173-8.

[2] L. Mikkelsen et al., “A global assessment of civil registration and vital statistics systems: monitoring data quality and progress,” The Lancet, vol. 386, no. 10001, pp. 1395–1406, Oct. 2015, doi: 10.1016/S0140-6736(15)60171-4.

[3] C. Lourenço Tavares de Andrade and C. Landmann Szwarcwald, “Desigualdades sócio-espaciais da adequação das informações de nascimentos e óbitos do Ministério da Saúde, Brasil, 2000-2002 Socio-spatial inequalities in the adequacy of Ministry of Health data on births and deaths at the municipal level in Brazil,” mai, 2007. [Online]. Available: http://www.datasus.

[4] A. L. Moncayo, G. Granizo, M. J. Grijalva, and D. Rasella, “Strong effect of Ecuador’s conditional cash transfer program on childhood mortality from poverty-related diseases: A nationwide analysis,” BMC Public Health, vol. 19, no. 1, Aug. 2019, doi: 10.1186/s12889-019-7457-y.

[5] D. Rasella, R. Aquino, and M. L. Barreto, “Impact of the Family Health Program on the quality of vital information and reduction of child unattended deaths in Brazil: an ecological longitudinal study,” 2010. [Online]. Available: http://www.biomedcentral.com/1471-2458/10/380

[6] D. M. Cavalcanti et al., “Evaluation and Forecasting Analysis of the Association of Conditional Cash Transfer With Child Mortality in Latin America, 2000-2030,” JAMA Netw Open, vol. 6, no. 7, p. e2323489, Jul. 2023, doi: 10.1001/jamanetworkopen.2023.23489.

[7] D. Rasella, R. Aquino, C. A. T. Santos, R. Paes-Sousa, and M. L. Barreto, “Effect of a conditional cash transfer programme on childhood mortality: A nationwide analysis of Brazilian municipalities,” The Lancet, vol. 382, no. 9886, pp. 57–64, 2013, doi: 10.1016/S0140-6736(13)60715-1.
